# Lipid-soluble smoke particles damage endothelial cells and reduce endothelium-dependent dilatation in rat and man

**DOI:** 10.1186/1471-2261-6-3

**Published:** 2006-01-19

**Authors:** Jin-Yan Zhang, Yong-Xiao Cao, Cang-Bao Xu, Lars Edvinsson

**Affiliations:** 1 Department of Pharmacology, Xi'an Jiaotong University School of Medicine, and Key Laboratory of Environment and Genes Related to Diseases (Xi'an Jiaotong University), Ministry of Education, Xi'an, Shaanxi 710061, P. R. China; 2 Division of Experimental Vascular Research, Institute of Clinical Science, Lund University, SE-22184, Sweden

## Abstract

**Background:**

Cigarette smoking is a strong risk factor for vascular disease and known to cause dysfunction of the endothelium. However, the molecular mechanisms involved are still not fully understood.

**Methods:**

In order to reveal the direct effects of lipid-soluble smoke particles on the endothelium, ring segments isolated from rat mesenteric arteries and human middle cerebral arteries (MCA) obtained at autopsy were incubated for 6 to 48 hrs in the presence of dimethylsulphoxide (DMSO)-soluble particles from cigarette smoke (DSP), i.e. lipid-soluble smoke particles. The endothelial microstructure was examined by transmission electron microscopy. The endothelial function was evaluated by acetylcholine (ACh)-induced endothelium-dependent vasodilatation, using a sensitive myograph.

**Results:**

After DSP treatment, the arterial endothelium was swollen and loosing its attachment. In functional tests, the total ACh-induced dilatation, the nitric oxide (NO)-mediated and the endothelium-derived hyperpolarization factor (EDHF)-mediated dilatations were significantly decreased by DSP in a time- and concentration-dependent manner (p < 0.05). Nicotine, an important compound in cigarette smoke had, in an equivalent concentration as in DSP, no such effects (p > 0.05). Similar results were obtained in the human MCA.

**Conclusion:**

Thus, we demonstrate that the lipid-soluble smoke particles, but not nicotine, caused damage to arterial endothelium and reduced the endothelium-dependent dilatation in man and rat.

## Background

Cigarette smoking has since long been recognized as a strong risk factor for the development of vascular disease such as hypertension, stroke and coronary heart disease [1,2]. Tobacco smoking is associated with increased intima-media thickness (IMT) of carotid and femoral arteries, elevated levels of circulating C-reactive protein (CRP), and correlates with atherosclerotic vascular disease. Although the inhaled smoke from the combustion of tobacco is known to cause development atherosclerotic vascular disease [3], however the mechanisms behind this are still elusive. Damage to endothelium-dependent vasodilatation induced by cardiovascular risk factors like cigarette smoke particles is believed to be involved in the development of vascular disease. Previously, we have demonstrated that dimethylsulfoxide (DMSO)-soluble particles from cigarette smoke (DSP), i.e. lipid-soluble smoke particles are toxic to cultured arterial endothelial (EC) and smooth muscle cells (SMC) [4,5]. A recent study showed that nicotine attenuates acetylcholine (ACh)-induced endothelium-dependent relaxation [6]. However, the role of nicotine in the development of cardiovascular disease is still being debated, since nicotine replacement by chewing tobacco or using moist snuff does not appear to have as many associated cardiovascular risks as smoking does [7].

Arterial endothelium possesses a number of important functions involved in regulation of vascular tone, proliferation and remodeling. Damage to the endothelium plays an important role in the development of atherosclerotic cardiovascular diseases [8]. We have previously demonstrated that organ culture of rat mesenteric arteries induces loss of nitric oxide (NO)- and prostaglandin-mediated vasodilatation, while this is compensated for by a stronger endothelium dependent hyperpolarization factor (EDHF) response, suggesting a shift of endothelium-mediated vasodilator mechanisms [9]. Cardiovascular risk factors like smoking may cause vascular disease via damage to endothelium-dependent vasodilatation.

The present study was designed, by using organ culture as a model, to investigate if lipid-soluble smoke particles cause damage to the endothelium and reduce the endothelium-dependent vasodilatation in man and in rat.

## Methods

### Reagents

Acetylcholine (ACh), 5-hydroxytryptamine (5-HT), N^G^-nitro-L-arginine methyl ester (L-NAME), apamin, charybdotoxin, indomethacin, nicotine, DMSO (Dimethyl Sulphoxide) and DMEM (Dulbecco's modified Eagle's medium) were purchased from Sigma, St. Louis, USA. All substances were dissolved in 0.9 % saline, except indomethacin and nicotine that were dissolved in DMSO.

### Extraction of DSP

Three cigarettes (0.8 mg nicotine per cigarette) were "smoked" by a water aspirator and the smoke was directed through a cotton wool filter. The retained smoke particles in the filter were dissolved in 1 ml DMSO. The DMSO-soluble smoke particle (DSP) preparations were analyzed by gas chromatograph-flame ionisation detection (GC-FID, Agilent 6890N, USA) with a 0.23 mm × 15 mm × 0.25 m DB-5MS capillary column (Agilent, USA). The GC-FID temperatures were programmed from 50°C, increased with 5°C/min to 280°C and remained for 3 min. The concentration of nicotine in DSP was calculated according to standard nicotine peak value and area. After DSP preparations have been analyzed by the gas chromatograph, they were diluted by DMSO to standard nicotine content (0.11 mg/L) and used for organ culture experiments.

### Tissue preparation

Sprague-Dawley rats (250~300 g, male or female) were euthanized with CO_2_, the head cut off and exsanguinated. The superior mesenteric artery was removed gently, immersed in cold oxygenated buffer solution (in mM: NaCl 119, NaHCO_3 _15, KCl 4.6, MgCl_2 _1.2, NaH_2_PO_4 _1.2, CaCl_2 _1.5, glucose 5.5, pH 7.4) and dissected free of adhering tissue under a light microscope. Human middle cerebral arteries (MCA) were obtained from 3 subjects from the Department of Forensic Medicine of Xian Jiaotong University (China). The arteries were collected from three men and used for organ culture between 4 to 6 hrs after death due to a traffic accident. The experimental protocol for using rat and human arteries was approved by local Ethic's Committee of Shaanxi province (China).

### Organ culture

The vessels were cut into 1 mm long cylindrical segments and placed in a 96-well plate, one segment in each well, containing 300 μl Dulbecco's modified Eagle's medium (DMEM) containing L-glutamine (584 mg/l) and supplemented with penicillin (100 U/ml) and streptomycin (100 μg/ml). The vessel segments were incubated for 6~48 h in the presence of DSP, the same volumes of DMSO as control or nicotine (0.11 mg/L) at 37°C in humidified 5 % CO_2_.

### *In vitro *pharmacology

The vessel segments were mounted on two L-shaped metal prongs. One prong was connected to a force displacement transducer (FT-03C) attached to a PowerLab (ADInstruments) unit for continuous recording of isometric force by means of the Chart software (ADInstruments). The other prong was connected to a displacement device, allowing fine adjustment of the distance between the two parallel prongs.

The mounted segments were immersed in tissue baths containing buffer solution, which was aerated continuously with a gas mixture of 95 % O_2 _and 5 % CO_2 _and maintained at 37°C. The artery segments were equilibrated for 1.5 h with a resting tension of 2.5 mN before the experiments were started. The contractile capacity of each vessel segment was examined by exposure to a K^+^-rich (60 mM) buffer solution in which NaCl was exchanged for an equimolar concentration of KCl. When two reproducible contractions had been achieved the vessels were used for further experiments.

After equilibration, the vessels were preconstricted with 5-HT (20 μM) or U46619 (1 μM). Once the sustained tension was obtained, ACh (10^-6 ^M) or cumulative ACh from 10^-10 ^to 10^-4 ^was added to the baths and the isometric tension was recorded. Vasodilatation was expressed as percentage of preconstriction with 5-HT or U46619. Nitric oxide (NO)-mediated dilatation induced by ACh was studied in the presence of 10 μM indomethacin, 50 nM charybdotoxin and 1 μM apamin. Endothelium-derived hyperpolarising factor (EDHF) was studied in the presence of 0.1 mM L-NAME and indomethacin. Prostaglandins (PG) were studied in the presence of L-NAME, charybdotoxin and apamin. The antagonists were added 20 minute before administration of 5-HT or U46619 preconstriction.

### Vascular microstructure

For studying vascular microstructure, a transmission electron microscopy (TEM, H-600, Hiatachi, Japan) was used. The fresh vessels were sectioned at 1 mm × 3 mm × 3 mm and fixed with 2.5 % Glutaraldehyde + 4 % Paraformaldehyde in phosphate buffer for 2 hours at 4°C, and washed with 0.1 M phosphate buffer for 30 min. The second fixation used was 1 % osmium tetroxide in phosphate buffer for 2 hours at 4°C, then washed with 2 – 3 changes of 0.1 M phosphate buffer, dehydrated with graded series of alcohol, immersed in mixture of propylene oxide + epon 812 for 1 hour or over night at 37°C. Then, they were exposed to a mixture of epon 812 for 1 hour at 60°C, embedded and polymerized for 48 hours at 60°C. Using Ultrathin Sectioning: LKB-V (LKB, Sweden) and sectioned at 50 – 70 nm, stained with uranyl acetate and lead hydroxide for 10 min, and subsequently examined in the transmission electron microscope.

### Calculation and statistics

The vasodilatation effects of ACh were expressed as percentage of preconstriction by agonists. Data were expressed as mean ± SEM. Student's *t*-test or two-way ANOVA with Bonferroni post-test was used to analyze difference between groups. The *P*-value of less than 0.05 was considered to be significant.

## Results

### Microstructure of artery endothelium

**Figure 1:**
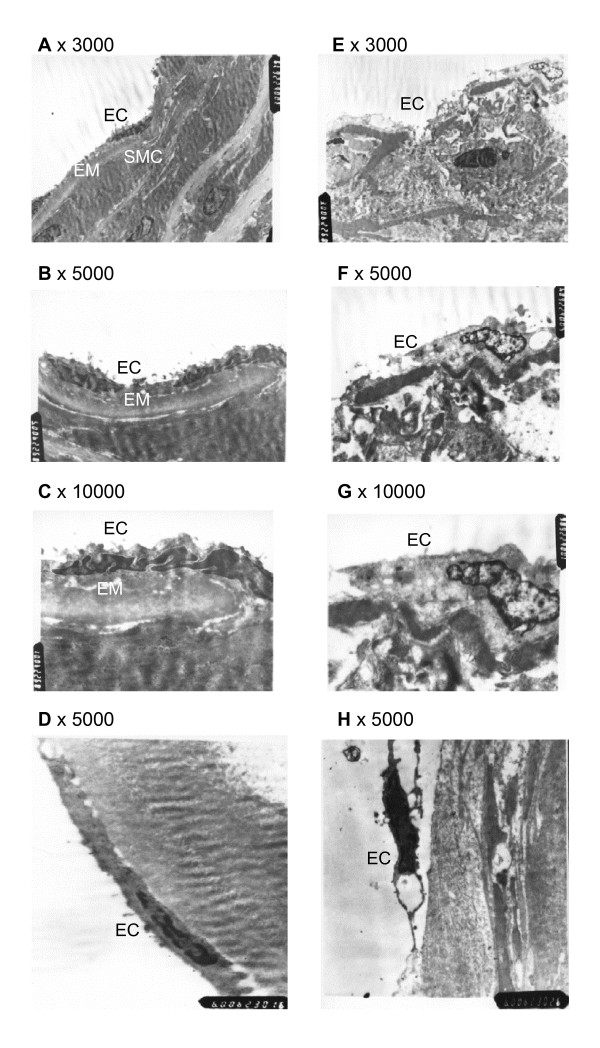
Endothelial microstructure. Rat mesenteric artery incubated with vehicle (DMSO 0.4 ml/L, A, B and C) or DSP (0.4 ml/L, E, F and G) for 24 hrs. Human MCA incubated with vehicle (DMSO 0.8 ml/L, D) or DSP (0.8 ml/l, H) for 12 hrs. Magnification: × 3000 for A and E; × 5000 for B, F, D and H; × 10000 for C and G. EC = endothelial cells; SMC = smooth muscle cells; EM = elastic membrane.

In control (DMSO) group, arterial endothelial cells were flat, clinging to the elastic membrane. There were junction structures between cells with microvillus-like lugs on the surface of the cell membranes. After exposure to DSP (0.4 ml/L) for 24 hrs, the endothelium was swollen and loose in rat mesenteric arteries (Fig. 1A-C and 1E-G), demonstrating that DSP caused damage to the arterial endothelium. In human middle cerebral arteries (MCA), after organ culture in the presence of DSP 0.8 ml/L for 12 h, similar damage to the endothelium was seen (Fig. 1D and 1H).

### Endothelium dependent dilations

**Figure 2:**
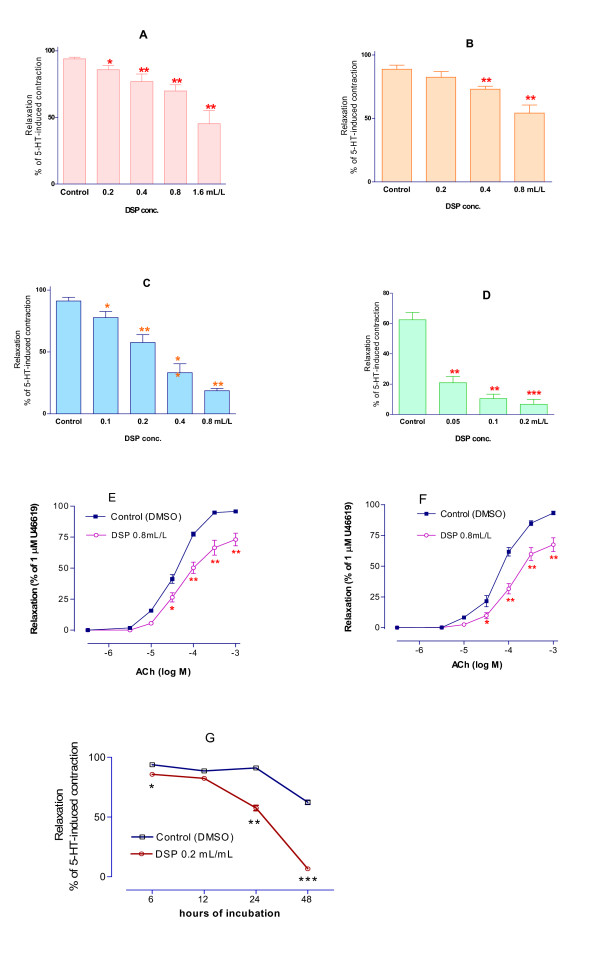
ACh-induced vasodilatation. The vasodilatation induced by ACh (10^-6 ^M) in the 5-HT (2 × 10^-5 ^M) preconstricted rat mesenteric artery segments cultured with DSP for 6 (A), 12 (B), 24 (C), 48 hrs (D) and the time course (G). ACh-induced vasodilatation in human MCA preconstricted by 1 μM of U46619 after treated with DSP 0.8 ml/L for 6 (E) and 12 hrs (F). Same volume of DMSO served as control. Data are derived from 8 segments and shown as mean ± S.E.M. *p < 0.05 and **p < 0.01 vs. control.

After 6 to 48 hrs of incubation with different concentrations of DSP (0.05–1.6 ml/L), the ACh-induced dilatation in rat mesenteric arterial segments was significantly attenuated over time (Fig. 2A-D and 2G), while in the control (DMSO) group, it was not significantly changed.

Human MCA segments were organ cultured for 6 or 12 hrs in the presence of DSP (0.8 ml/L). Similar results were obtained as in rat mesenteric arteries with a reduction of the ACh-induced vasodilatation (Fig. 2E-F).

### EDHF-mediated dilatations

**Figure 3:**
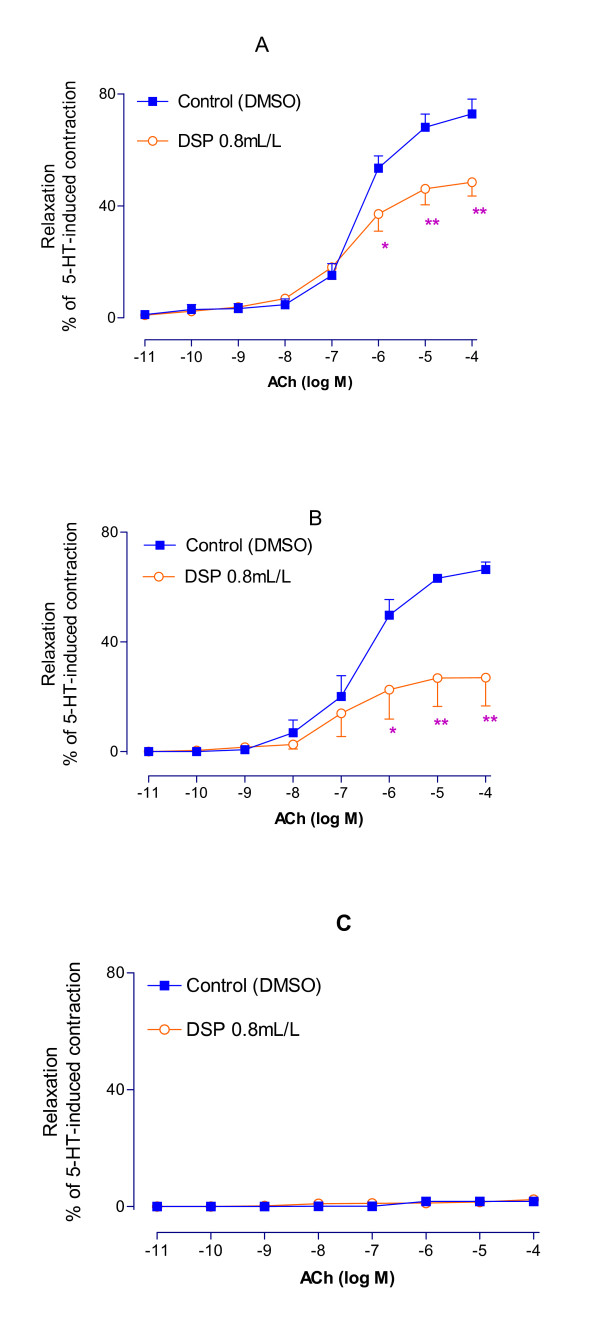
Characterizations of ACh-induced vasodilatation. (A) Changes in ACh-induced EDHF mediated vasodilatations on the 5-HT preconstricted rat mesenteric arteries after treated with DSP for 12 hours. The tests were performed in the presence of indomethacin (10^-5 ^M) and L-NAME (10^-4 ^M). (B) Changes in ACh-induced NO mediated vasodilatations on the 5-HT preconstricted rat mesenteric arteries after treated with DSP for 12 hours. The tests were done in the presence of indomethacin, charybdotoxin (5 × 10^-8 ^M) and apamin (10^-6 ^M). (C) Changes in ACh-induced PG mediated vasodilatations on the 5-HT preconstricted rat mesenteric arteries after treated with DSP for 12 hours. The tests were done in the presence of charybdotoxin, apamin and L-NAME. Each data points derived from 8 experiments and shown as mean ± S.E.M. *p < 0.05 and **p < 0.01 vs. control.

To study EDHF-mediated dilatation, the vessel segments were incubated for 12 hrs with DSP (0.8 ml/L), precontracted with 5-HT (2 × 10^-5 ^M) and relaxed by cumulative application of ACh (10^-10^~10^-4 ^M) in the presence of L-NAME (10^-4 ^M) and indomethacin (10^-5 ^M). The maximal dilatation was significantly lower in DSP-treated than in the control (DMSO) group (*P *< 0.01) (Fig. 3A).

### NO-mediated dilatations

To study NO-mediated dilatation, indomethacin (10^-5 ^M), charybdotoxin (5 × 10^-8 ^M) and apamin (10^-6 ^M) were used to block prostaglandin and EDHF mediated effects. The experiments were otherwise performed as above. The maximal NO-mediated dilatation was significantly reduced in DSP-treated segments as compared to the control group (*P *< 0.01) (Fig. 3B).

### PG-mediated dilatations

The vessel segments were studied as above but in the presence of L-NAME (10^-4 ^M), charybdotoxin (5 × 10^-8 ^M) and apamin (10^-6 ^M) to block NO- and EDHF-mediated relaxation. The maximal PG-dilatation was slight or near nil. There were no observable differences between the control and the DSP treated groups (Fig. 3C).

### Comparison of DSP with nicotine

**Figure 4:**
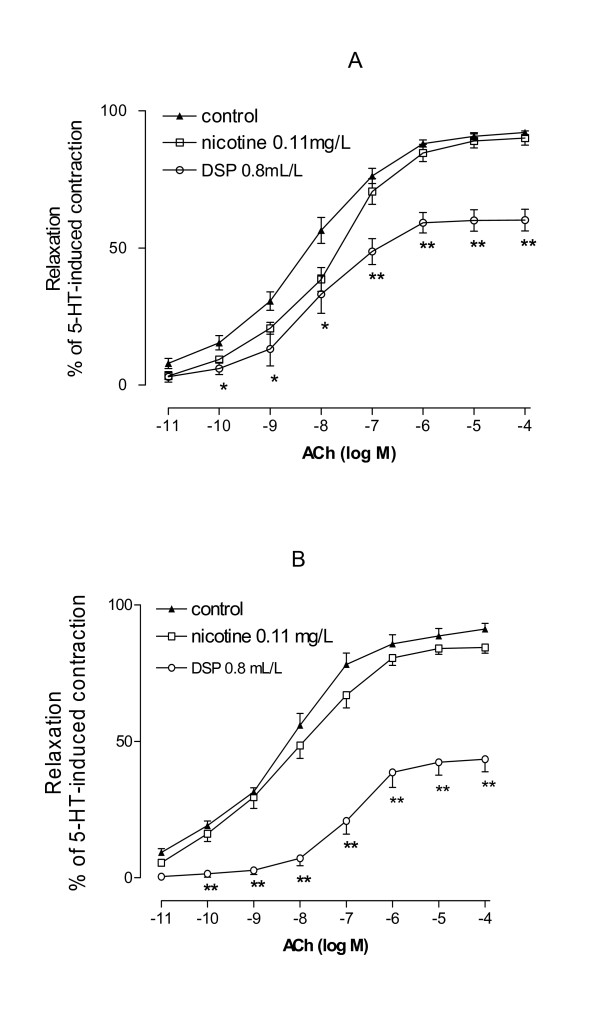
Effects of nicotine in comparison with DSP on ACh-induced vasodilatation. Concentration 0.11 mg/L of nicotine that is nicotine concentration in DSP was used. Since nicotine and smoke particles were dissolved in DMSO, the same volume of DMSO was added in the culture medium and served as controls. The arteries treated with nicotine or DSP for 6 (A) and 12 hrs (B). Data are shown as mean ± S.E.M. n = 8. *p < 0.05 and **p < 0.01 vs. control.

DSP contains 0.11 mg/L of nicotine and thus this concentration was used to examine if nicotine was the key substance in the DSP-induced reduction of vasodilatation. Compared to nicotine, DSP had a significantly stronger effect in reducing the ACh-induced dilatation. However, in comparison with DMSO (control) nicotine did not produce any significant reduction of vasodilatation (Fig. 4).

## Discussion

The present study has revealed that lipid-soluble cigarette smoke particles "DSP", but not nicotine in equivalent concentration, caused damage to the artery endothelium and reduced the endothelium-dependent relaxation in rat mesenteric artery and in human MCA. This agrees well with previous findings since DSP has been found to cause damage to cultured arterial EC and SMC cells [4]. The endothelium has many important functions such as regulating arterial tone, antithrombosis, release of growth factors and growth inhibitors as well as expressing vascular adhesion molecules [4,8]. Studies in active and passive smoking subjects have shown that they have impaired endothelial function [10]. According to the "response to injury" hypothesis of atherosclerosis, dysfunction of endothelium is an early event in the development of atherosclerotic vascular disease. Thus, the present study unveils a link between cigarette smoking and vascular disease, since lipid-soluble smoke particles (DSP) caused endothelial damage and dysfunction. However, nicotine, an important compound in cigarette smoking, in an equivalent concentration as in DSP, did not show such effects.

The ACh-induced endothelium-dependent relaxation consists of NO-, PG- and EDHF-mediated vasodilatation [9]. NO is generated by nitric oxide synthase (NOS) and inhibited by the NOS inhibitor L-NAME [11]. This results in relaxation of vascular smooth muscle by activating guanylate cyclase. Cyclo-oxygenase (COX) converts arachidonic acid to prostaglandins that relax vascular smooth muscle by activating adenylate cyclase. Prostaglandin formation can be inhibited by a general COX inhibitor indomethacin. EDHF is distinct from NO and prostaglandins, which hyperpolarizes and relaxes smooth muscle cells. Both its vasodilator and hyperpolarizing effects can be inhibited by a combination of potassium channel inhibitor charybdotoxin and apamin [12]. L-NAME, indomethacin, charybdotoxin and apamin were used in the present study to define the pathway of endothelium-dependent relaxation [9] altered by DSP. The results show that reduction of NO- and EDHF-mediated vasodilatation by DSP occurred together with damage to the endothelial microstructure, while PG-mediated vasodilatation was not altered. NO and EDHF are important vasodilator substances derived from the endothelium. Damage to NO- and EDHF-mediated endothelium-dependent relaxations by lipid-soluble smoke particles is favorable to vasoconstrictor domination in vascular disease such as hypertension. Epidemiological studies have shown that smoking and hyperlipidemia are synergistic risk factors [1]. We have previously demonstrated that DMSO-soluble particles from cigarette smoke are directly toxic to cultured arterial endothelial and smooth muscle cells [4,5]. Lipid-soluble smoke particles in blood are transported by lipoproteins to the arterial wall [13] and thus may directly impair the function of endothelial cells or cause damage to the arterial wall via modification of lipoprotein properties [5].

Nicotine replacement by chewing tobacco or using moist snuff appears as an opportunity to reduce tobacco smoking-related cardiovascular harm since most smokers inhale smoke in order to obtain a rapid rise in the nicotine level in blood. Smoke-free tobacco users who chew tobacco or take moist snuff have lower risk for cardiovascular disease than tobacco smokers [14]. In the WHO cardiovascular risk factor project in Sweden two case-control studies have shown no increase in risk for myocardial infarction by using moist snuff [15]. Theses studies suggest that tobacco is more harmful when it is smoked. Chewing tobacco or application of moist snuff gets water soluble particles into the blood while smoking in addition leads the lipid-soluble particles through the lung to enter the blood. Different properties of particles from tobacco may explain the different effects on cardiovascular disease between chewing tobacco and inhaling smoked tobacco. In the present study, we have shown that lipid-soluble smoke particles caused damage to the arterial endothelium, while nicotine in an equivalent concentration as in DSP, did not alter endothelium-dependent vasodilatation. This suggests that it is not nicotine per se in DSP that caused the reduction of vasodilatation. However, nicotine has been reported to cause damage to endothelium-dependent relaxation [6]. The different experimental protocols, species and arteries used may explain the difference.

## Conclusion

We have here demonstrated that lipid-soluble smoke particles, but not nicotine, cause damage to the arterial endothelium and reduced the endothelium-dependent dilatation in man and rat.

## Competing interests

The author(s) declare that they have no competing interests.

## Authors' contributions

ZIY: carried out the studies and wrote the first draft of the manuscript.

CYX: participated in the design of the study and performed the statistical analysis.

CBX: conceived the study, and participated in its design, coordination and helped to draft the manuscript.

LE: participated in its design and revised the manuscript.

All authors have read and approved the final manuscript.

## Pre-publication history

The pre-publication history for this paper can be accessed here:


